# Supplementary material for: Content-rich biological network constructed by mining PubMed abstracts
Source: BMC Bioinformatics. 2004 Oct 8;5:147. doi: 10.1186/1471-2105-5-147 (PMC528731; doi:10.1186/1471-2105-5-147)
Supplement: Additional File 2 — The original results of the above study (non-essential files are deleted to keep the file size under the limit set by BMC bioinformatics). [file 1471-2105-5-147-S2.bz2 › chilibotAdditionalFile2/dip30/583ID9533885E10951/html/NUTF2_RAN.html]

 


 **NUTF2** and **RAN** 
  
Found 47 abstracts in PubMed, retrieved 30.  
 

 What does Google say? 
 PDF only 
| .edu only 

---

**Interactive relationship** (e.g. stimulation, inhibition, etc)

**Stimulatory relationship**- An NTF2  [ **NUTF2** ]  mutant that cannot bind  **Ran**  6 was unable to facilitate  **Ran**  import.  Ref: 9889103 Curr Biol< MedlineTA>Curr Biol,
- Recently, it has been reported that NTF2  [ **NUTF2** ]  can stimulate the accumulation of  **Ran**  in digitonin permeabilized cells.  Ref: 10679025 Mol Biol Cell, 2000
- Monoclonal antibodies to NTF2  [ **NUTF2** ]  inhibit nuclear protein import by preventing nuclear translocation of the GTPase  **Ran** .  Ref: 10679025 Mol Biol Cell, 2000
- These results indicate that, instead,  **Ran**  increases the binding of NTF2  [ **NUTF2** ]  to nucleoporins by another mechanism, most probably by  **Ran**  itself binding to nucleoporins and NTF2  [ **NUTF2** ]  binding to this nuclear pore associated  **Ran** .  Ref: 10681579 J Biol Chem, 2000
- In this system, export is dependent on exogenous CRM1 Exportin1 a nuclear export receptor the GTPase  **Ran**  and nucleotide triphosphates NTPs, and is further stimulated by  **Ran**  binding protein 1 RanBP1 and nuclear transport factor 2  [ **NUTF2** ]  NTF2  [ **NUTF2** ] .  Ref: 9889120 Curr Biol, 1999
- Moreover, anti NTF2  [ **NUTF2** ]  mAbs inhibited nuclear import of both  **Ran**  and NLS containing protein in vitro, suggesting that NTF2  [ **NUTF2** ]  stimulates NLS dependent protein import by driving the nuclear accumulation of  **Ran** .  Ref: 10679025 Mol Biol Cell, 2000
**Neutral relationship**- While transport factors such as  **Ran**  and NTF2  [ **NUTF2** ]  show only transient interaction with the pores, a number of soluble transport receptors, including importin beta, show a tight association with the purified pores.  Ref: 11208084 Traffic, 2000
- The interaction between  **Ran**  and NTF2  [ **NUTF2** ]  is required for cell cycle progression.  Ref: 10930458 Mol Biol Cell, 2000
- This provides the first evidence that NTF2  [ **NUTF2** ]  regulates the distribution of  **Ran**  in vivo.  Ref: 10679025 Mol Biol Cell, 2000
- Identification of an NTF2  [ **NUTF2** ]  related factor that binds  **Ran**  GTP and regulates nuclear protein export.  Ref: 10567585 Mol Cell Biol, 1999
- These findings demonstrate that NTF2  [ **NUTF2** ]  is required to transport  **Ran**  into the nucleus in vivo.  Ref: 10930458 Mol Biol Cell, 2000
- A temperature sensitive ts mutant of Saccharomyces cerevisiae NTF2  [ **NUTF2** ]  that does not bind to  **Ran**  is unable to import  **Ran**  into the nucleus at the nonpermissive temperature.  Ref: 10930458 Mol Biol Cell, 2000
- The interaction between NTF2  [ **NUTF2** ]  and  **Ran**  is essential for the import of  **Ran**  into the nucleus.  Ref: 11489893 J Biol Chem, 2001
- A GTP bound form of a  **Ran**  mutant that cannot bind NTF2  [ **NUTF2** ]  was not a substrate for import.  Ref: 9889103 Curr Biol< MedlineTA>Curr Biol, 2001
- Nuclear import of  **Ran**  is mediated by the transport factor NTF2  [ **NUTF2** ] .  Ref: 9889103 Curr Biol< MedlineTA>Curr Biol, 2001
- Here we show that cytoplasmic injection of anti NTF2  [ **NUTF2** ]  mAbs resulted in a dramatic relocalization of  **Ran**  to the cytoplasm.  Ref: 10679025 Mol Biol Cell, 2000
- The addition of energy and nuclear transport factor 2  [ **NUTF2** ]  NTF2  [ **NUTF2** ]  5 was sufficient for the accumulation of  **Ran**  in the nucleus.  Ref: 9889103 Curr Biol< MedlineTA>Curr Biol, 2000
- We show that nuclear transport factor 2  [ **NUTF2** ]  NTF2  [ **NUTF2** ]  is required to concentrate  **Ran**  in the nucleus in the budding yeast, Saccharomyces cerevisiae.  Ref: 10889207 J Biol Chem, 2000
- NTF2  [ **NUTF2** ]  mediated import of  **Ran**  appears to be the bottleneck for maximal capacity of  **Ran**  driven nuclear transport.  Ref: 12606574 EMBO J, 2003
- Evidence that NTF2  [ **NUTF2** ]  directly mediates  **Ran**  import or that NTF2  [ **NUTF2** ]  is required to maintain the nuclear concentration of  **Ran**  in living cells has not been obtained.  Ref: 10679025 Mol Biol Cell, 2000
- Taken together, our data show that NTF2  [ **NUTF2** ]  is an essential regulator of the  **Ran**  distribution in living cells.  Ref: 10679025 Mol Biol Cell, 2000
- This interaction between NTF2  [ **NUTF2** ]  and GDP  **Ran**  is required for efficient nuclear protein import and may function between the docking and translocation steps of the pathway.  Ref: 9878368 J Mol Biol, 1998
- We used the crystal structure of the GDPRan NTF2  [ **NUTF2** ]  complex to design mutants in the switch II loop of  **Ran**  to probe the contribution of Lys71, Phe72 and Arg76 to this interaction.  Ref: 10356329 J Mol Biol, 1999
- Nuclear protein import requires a precisely choreographed series of interactions between nuclear pore components and soluble factors such as importin beta,  **Ran** , and nuclear transport factor 2  [ **NUTF2** ]  NTF2  [ **NUTF2** ] .  Ref: 10356329 J Mol Biol, 1999
- **Ran**  function is modulated in a noncatalytic fashion by NTF2  [ **NUTF2** ] , a protein that mediates nuclear import of  **Ran**  GDP.  Ref: 10567585 Mol Cell Biol, 1999
- NTF2  [ **NUTF2** ]  has been shown to bind nuclear pore complex proteins and the GDP form of  **Ran**  in vitro.  Ref: 10679025 Mol Biol Cell, 2000
- Here we characterize a novel component of the  **Ran**  system that is 26% identical to NTF2  [ **NUTF2** ] , which based on its function we refer to as NTF2  [ **NUTF2** ]  related export protein 1 NXT1.  Ref: 10567585 Mol Cell Biol, 1999
- Engineered mutants in the switch II loop of  **Ran**  define the contribution made by key residues to the interaction with nuclear transport factor 2  [ **NUTF2** ]  NTF2  [ **NUTF2** ]  and the role of this interaction in nuclear protein import.  Ref: 10356329 J Mol Biol, 1999
- Proteins that regulate the GTPase cycle and subcellular distribution of  **Ran**  include the cytoplasmic GTPase activating protein RanGAP and its co factors RanBP1, RanBP2, the nuclear guanine nucleotide exchange factor RanGEF, and the  **Ran**  import receptor NTF2  [ **NUTF2** ] .  Ref: 10811801 J Biol Chem, 2000
- Engineered mutants at the interaction interface reduce the binding of NTF2  [ **NUTF2** ]  to nuclear pores and cause reduced growth rates and  **Ran**  mislocalization when substituted for the wild type protein in yeast.  Ref: 12065398 EMBO J, 2002

**Non-interactive relationship** (e.g. studied together, co-existance, homology, etc.)

- In contrast to NTF2  [ **NUTF2** ] , NXT1 preferentially binds  **Ran**  GTP, and it colocalizes with the nuclear pore complex NPC in mammalian cells.  Ref: 10567585 Mol Cell Biol, 1999
- We conclude that NTF2  [ **NUTF2** ]  functions as a transport receptor for  **Ran** , permitting rapid entry into the nucleus where GTP GDP exchange mediated by RCC1 7 converts  **Ran**  into its GTP bound state.  Ref: 9889103 Curr Biol< MedlineTA>Curr Biol, 1999
- Here, we examine the in vivo role of NTF2  [ **NUTF2** ]  in  **Ran**  import and the effect that disruption of  **Ran**  imported into the nucleus has on the cell cycle.  Ref: 10930458 Mol Biol Cell, 2000
- The TAP p15 heterodimer is structurally similar to the homodimeric transport factor NTF2  [ **NUTF2** ] , but unlike NTF2  [ **NUTF2** ] , it is incompatible with either homodimerization or  **Ran**  binding.  Ref: 11583626 Mol Cell, 2001
- Consistent with a critical role for NTF2  [ **NUTF2** ]  in establishing and maintaining the  **Ran**  gradient, we show that NTF2  [ **NUTF2** ]  is required for early embryogenesis in Caenorhabditis elegans.  Ref: 10889207 J Biol Chem, 2000
- Four mutant complementation groups were characterized that correspond to respective mutations in genes encoding  **Ran**  gsp1, and essential  **Ran**  regulatory factors  **Ran**  GTPase  [ **RAN** ]  activating protein rna1,  **Ran**  guanine nucleotide exchange factor prp20, and the RanGDP import factor ntf2  [ **NUTF2** ] .  Ref: 12654904 J Cell Biol, 2003
- The GDP bound form of the switch II mutants showed no detectable binding to NTF2  [ **NUTF2** ] , providing direct evidence that salt bridges involving Lys71 and Arg76 and burying Phe72 are all crucial for the interaction between  **Ran**  and NTF2  [ **NUTF2** ] .  Ref: 10356329 J Mol Biol, 1999
- ... addition of transportin, which accumulates in the nucleus, enhanced NTF2  [ **NUTF2** ]  dependent  **Ran**  import.  Ref: 9889103 Curr Biol< MedlineTA>Curr Biol, 1999
- To analyze the mechanism of  **Ran**  import into the nucleus by NTF2  [ **NUTF2** ] , we use mutants in a variety of nuclear transport factors along with biochemical analyses of NTF2  [ **NUTF2** ]  complexes.  Ref: 10889207 J Biol Chem, 2000
- This conformational change alters the positions of key residues such as Lys71, Phe72 and Arg76 that are crucial for the interaction of GDP  **Ran**  with NTF2  [ **NUTF2** ]  and indeed, solution binding studies were unable to detect any interaction between NTF2  [ **NUTF2** ]  and GDP RanQ69L under conditions where GDP  **Ran**  bound effectively.  Ref: 9878368 J Mol Biol, 1998
- When the structure of GDP RanQ69L from monoclinic crystals with P21 symmetry was compared with the structure of wild type  **Ran**  obtained from monoclinic crystals, the Q69L mutant showed a large conformational change in residues 68 74, which are in the switch II region of the molecule which changes conformation in response to nucleotide state and which forms the major interaction interface with nuclear transport factor 2  [ **NUTF2** ]  NTF2  [ **NUTF2** ] , sometimes called p10.  Ref: 9878368 J Mol Biol, 1998
- NLS dependent nuclear import was reconstituted in vitro using the different importin subunits together with the transport factors  **Ran**  and NTF2  [ **NUTF2** ] .  Ref: 10336485 J Biol Chem, 1999
- In the permeabilized cell in vitro transport system, nuclear import of SREBP 2 is reconstituted only by importin beta in conjunction with  **Ran**  and its interacting protein p10 NTF2  [ **NUTF2** ] .  Ref: 10397761 Mol Biol Cell, 1999
- NTF2  [ **NUTF2** ]  mediated  **Ran**  nuclear import is required for NLS dependent protein import.  Ref: 10679025 Mol Biol Cell, 2000
- We find that  **Ran**  remains concentrated in the nucleus when importin mediated protein import is disrupted and demonstrate that NTF2  [ **NUTF2** ]  does not form a stable complex with the transport receptor, importin beta.  Ref: 10889207 J Biol Chem, 2000
- The mechanism of  **ran**  import into the nucleus by nuclear transport factor 2  [ **NUTF2** ] .  Ref: 10889207 J Biol Chem, 2000
- Although the accumulation of NTF2  [ **NUTF2** ]  at the nuclear envelope is enhanced by RanGDP, we show that  **Ran**  binding does not alter the affinity of NTF2  [ **NUTF2** ]  for nucleoporins nor does the binding of nucleoporins alter the affinity of NTF2  [ **NUTF2** ]  for RanGDP.  Ref: 10681579 J Biol Chem, 2000
- Our data distinguish between two possible mechanisms for  **Ran**  import by NTF2  [ **NUTF2** ]  and demonstrate that  **Ran**  import is independent from importin beta mediated protein import.  Ref: 10889207 J Biol Chem, 2000
- In contrast, a significant reduction in affinity of the RanGDP binding protein NTF2  [ **NUTF2** ]  for  **Ran**  CoreGDP is observed, indicating that the switch regions have changed conformation in the  **Ran**  Core mutant.  Ref: 12051861 J Mol Biol, 2002
- In rat liver nuclei, poly A RNA, hnRNPs A1 and C, mrnp 41, ASF, and a phosphorylated subset of SR proteins were also distributed like mRNA, as were various transport factors and their cargoes NTF2  [ **NUTF2** ] ,  **Ran** , RCC1, karyopherin beta, Rch1, transportin alpha, m, 7 trimethylG.  Ref: 10633080 J Cell Sci, 2000
- The structure of the Q69L mutant of GDP  **Ran**  shows a major conformational change in the switch II loop that accounts for its failure to bind nuclear transport factor 2  [ **NUTF2** ]  NTF2  [ **NUTF2** ] .  Ref: 9878368 J Mol Biol, 1998
- Surprisingly, we found that neither Xenopus ovarian cytosol nor a mixture of recombinant import factors karyopherin alpha2, karyopherin beta1,  **Ran** , and p10 NTF2  [ **NUTF2** ]  were able to support the import of pA RCC1 into the nuclei of digitonin permeabilized cells.  Ref: 10744690 J Biol Chem, 2000
- Nuclear protein accumulation in digitonin permeabilzed cells was impaired with  **Ran**  mutants deficient in NTF2  [ **NUTF2** ]  binding, confirming that the NTF2  [ **NUTF2** ]   **Ran**  interaction is required for efficient transport.  Ref: 10356329 J Mol Biol, 1999
- 4 Importin beta1,  **Ran** , and p10 NTF2  [ **NUTF2** ]  are sufficient to mediate import of activated Smad3.  Ref: 11294908 Mol Biol Cell, 2001
- We have used a range of complementary biochemical and biophysical methods to investigate the interactions between nuclear transport factor 2  [ **NUTF2** ]  NTF2  [ **NUTF2** ] , the Ras family GTPase  **Ran** , and XFXFG nucleoporin repeats that are crucial for nuclear trafficking.  Ref: 10681579 J Biol Chem, 2000
